# Radiographic Anatomy and Clinical Value of the Modified Corner Approach in Interlaminar Endoscopic Lumbar Discectomy

**DOI:** 10.1111/os.70143

**Published:** 2025-07-31

**Authors:** Sizheng Zhan, Haoning Ma, Yuming Wang, Ping Yi, Xiangsheng Tang

**Affiliations:** ^1^ Department of Spine Surgery China Japan Friendship Hospital Beijing China

**Keywords:** corner approach, IELD, learning curve, radiographic anatomy

## Abstract

**Objective:**

The first step of interlaminar endoscopic lumbar discectomy (IELD) is puncture localization, which lacks standardized protocols and requires a significant learning curve. To address this, we developed a modified corner approach targeting the junction of the S1 superior endplate and facet joint. This study aims to characterize the radiographic anatomy and assess the clinical utility of this modified approach.

**Method:**

Computed tomography (CT) and magnetic resonance imaging (MRI) data from 100 patients were analyzed to measure distances between the target and adjacent structures (dura sac, pedicle, L5 nerve, and S1 nerve). The learning curve of interlaminar endoscopic lumbar discectomy (IELD) surgery based on the modified corner approach was determined by prospectively collecting data from 80 patients.

**Results:**

The mean distance between the target and the dura sac was 4.59 ± 1.74 mm. The mean distance between the target and the inferior border of the L5 nerve was 10.14 ± 1.72 mm, rang from 7.52 to 13.54 mm. The mean distance between the target and the outer edge of the S1 nerve was 0.51 ± 0.91 mm, rang from −0.12 to 2 mm. The mean distance between the target and the inner edge of the S1 pedicle was 3.77 ± 1.04 mm. The distance between the target and the dura sac and the inner edge of the pedicle is mainly affected by the patient's age.

**Conclusion:**

The modified corner approach is a simple, safe, and repeatable surgical approach with the intersection of the superior endplate and facet joint as the puncture target. For patients without or with mild facet joint degeneration, the puncture target can be appropriately moved inward by 2 mm.

## Graphical abstract

The modified corner approach is a simple, standard, and repeatable surgical approach with the intersection of the superior endplate and facet joint as the puncture target. For patients without or with mild facet joint degeneration, the puncture target can be appropriately moved inward by 2 mm.

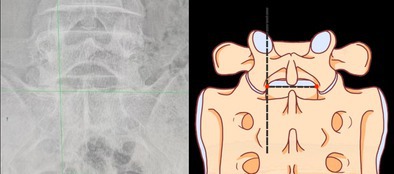

## 1 Introduction

Lumbar disc herniation (LDH) is one of the most common spinal disorders predominantly affecting lower lumbar nerve roots [1]. Treatment is initially conservative. Persistent limb or lumbar pain or neurologic deficits may lead to consideration of surgical interventions and removal of intervertebral disc [2].

As a minimally invasive procedure, full endoscopic lumbar discectomy (FELD) or percutaneous endoscopic lumbar discectomy (PELD) is as efficacious as the traditional open discectomy, yet has a number of advantages over the open procedure, including less paravertebral muscle injury, shorter hospital stay, and quicker functional recovery [3]. Although there are many variations of the approach, most fall into two categories: transforaminal endoscopic lumbar discectomy (TELD) and interlaminar endoscopic lumbar discectomy (IELD) [4]. Due to the traditional posterior approach and larger operational space, ILED is currently more widely used in clinical practice [5], especially for L5/S1.

Compared with the traditional open discectomy, IELD is prone to be disoriented during the operation [5]. It is difficult to judge the position in time and accurately, either inside or outside the spinal canal, which may lead to more operative time, increased surgical risks, and even the possibility of conversion to open surgery [5], especially for beginners [6]. In a recent meta‐analysis, Ahn et al. [7] found that the average learning period of IELD was 22 operations, and the differences were mainly the operation time and surgical complications, mainly due to the unfamiliarity of intraoperative anatomy.

The first step of IELD is puncture localization [8]. The targets include ligamentum flavum [9], lamina [10] or facet joint [11] without a uniform standard. Among them, there is an approach called the corner approach, where the corner refers to the transitional area between the upper margin of the lamina and the superior facet joint. However, the corner approach is inconsistent among surgeons because the corner is an area and is not well defined on the X‐ray.

Based on the corner approach, we designed a modified corner approach and the intersection of the S1 superior endplate and the facet joint was selected as the target or indicator. Based on the patient's radiological imaging data and a series of clinical operations, we intend to: (1) obtain the critical anatomical features applicable to the modified corner approach; (2) verify the safety and effectiveness of the modified corner approach in IELD.

## 2 Method

### 2.1 Modified Corner Approach

**FIGURE 1:**
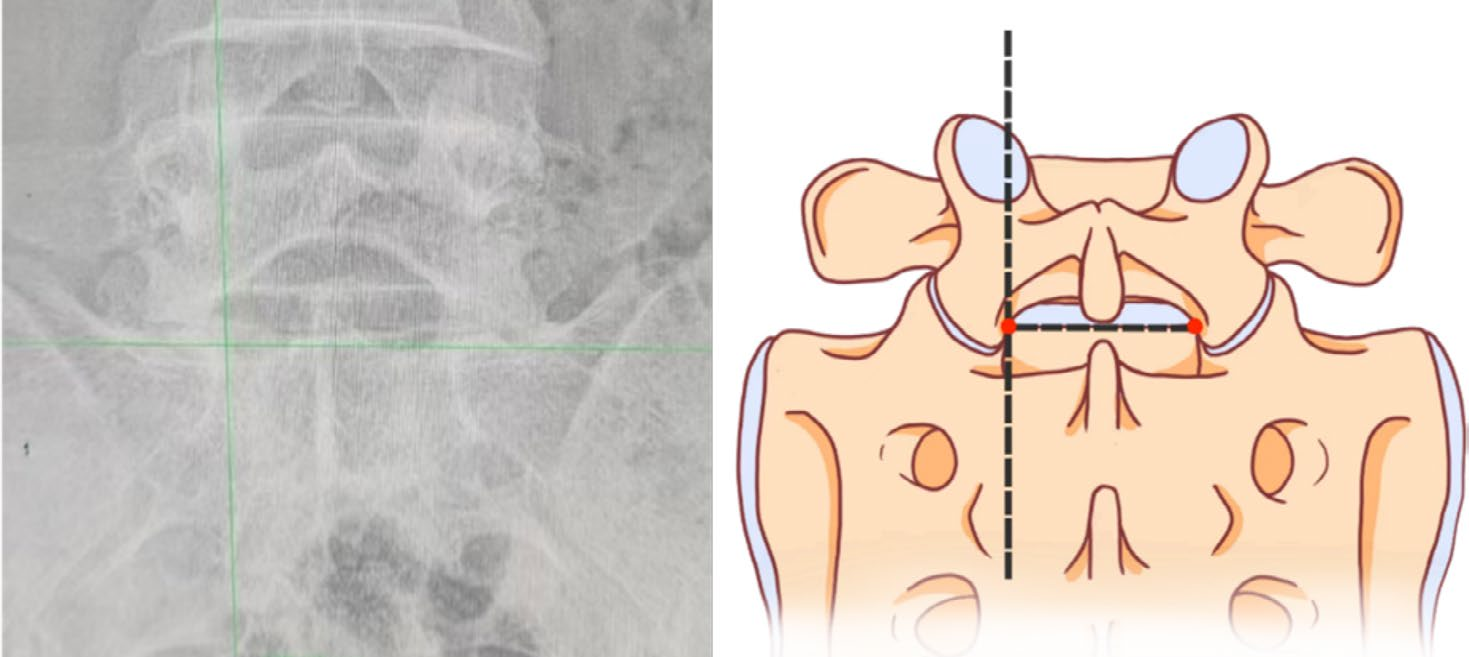
The intersection of the S1 superior endplate and the facet joint was taken as the puncture target.

In the anteroposterior view of the patient, the intersection of the S1 superior endplate and the facet joint was taken as the puncture target (Figure 1). A 1 cm longitudinal incision was made. The pencil tip is placed vertically until the hard bone surface. The working channel is then inserted We hope that an ideal access can meet the following conditions as far as possible: (1) Safety. Anatomical landmarks could be recognized in time during the whole operation. (2) Simplicity. The operation process is standardized and repeatable; (3) Convenience. The operation is convenient, including puncture and discectomy.

### 2.2 Population for Obtaining the Radiographic Anatomical Features

Prior to the initiation of the study, our study protocol was approved by the Ethics Committee of China‐Japan Friendship Hospital (Approval No. 2024‐KY‐263). Informed consent was obtained from all participants prior to their inclusion in the study. This study was conducted in accordance with the principles of the Declaration of Helsinki. A total of 100 patients with low back pain and other reasons who underwent both 3‐dimensional (3D) lumbar computed tomography (CT) and magnetic resonance imaging (MRI) in China‐Japan Friendship Hospital from 2024.1 to 2024.10 were retrospectively enrolled. The inclusion criteria were: (1) 18–80 years old, regardless of gender; (2) completed both the 3D lumbar CT examination and MRI. Exclusion criteria: (1) history of lumbar surgery; (2) lumbar spondylolisthesis; (3) scoliosis with Cobb angle > 5°; (4) history of lumbar fracture; (5) nerve compression due to degeneration or LDH.

### 2.3 Population for Assessing the Clinical Value

At the same time, a total of 80 patients with LDH who underwent IELD surgery from 2024.1 to 2024.10 were retrospectively included to verify the safety and clinical significance of the modified corner approach. But the data is collected from a prospective database. The inclusion criteria were: (1) 18–80 years old, regardless of gender; (2) L5/S1 lumbar disc herniation; (3) IELD surgery is indicated. Exclusion criteria: (1) lumbar spondylolisthesis; (2) lumbar spinal stenosis; (3) scoliosis with Cobb angle > 5°.

### 2.4 Imaging Analysis

The images in this study were from a CT machine (SOMATOM Definition Flash from SEMENS.) and a 3 T MR scanner (Ingenia; Philips Healthcare, Best, the Netherlands). All measurements were taken based on the 3D reconstructed lumbar CT images and MRI using the PACS. All quantitative measurements were analyzed using the mean values of the measurements obtained by the two investigators independently. **a** refers to the transverse diameter of the intersection point of the two sides (based on CT data), **b** refers to the transverse diameter of the dura mater at the same level (based on MRI), and **c** refers to the distance between the outer edges of the S1 nerve at the same level (based on MRI).

#### 2.4.1 Dural Sac Distance

**FIGURE 2:**
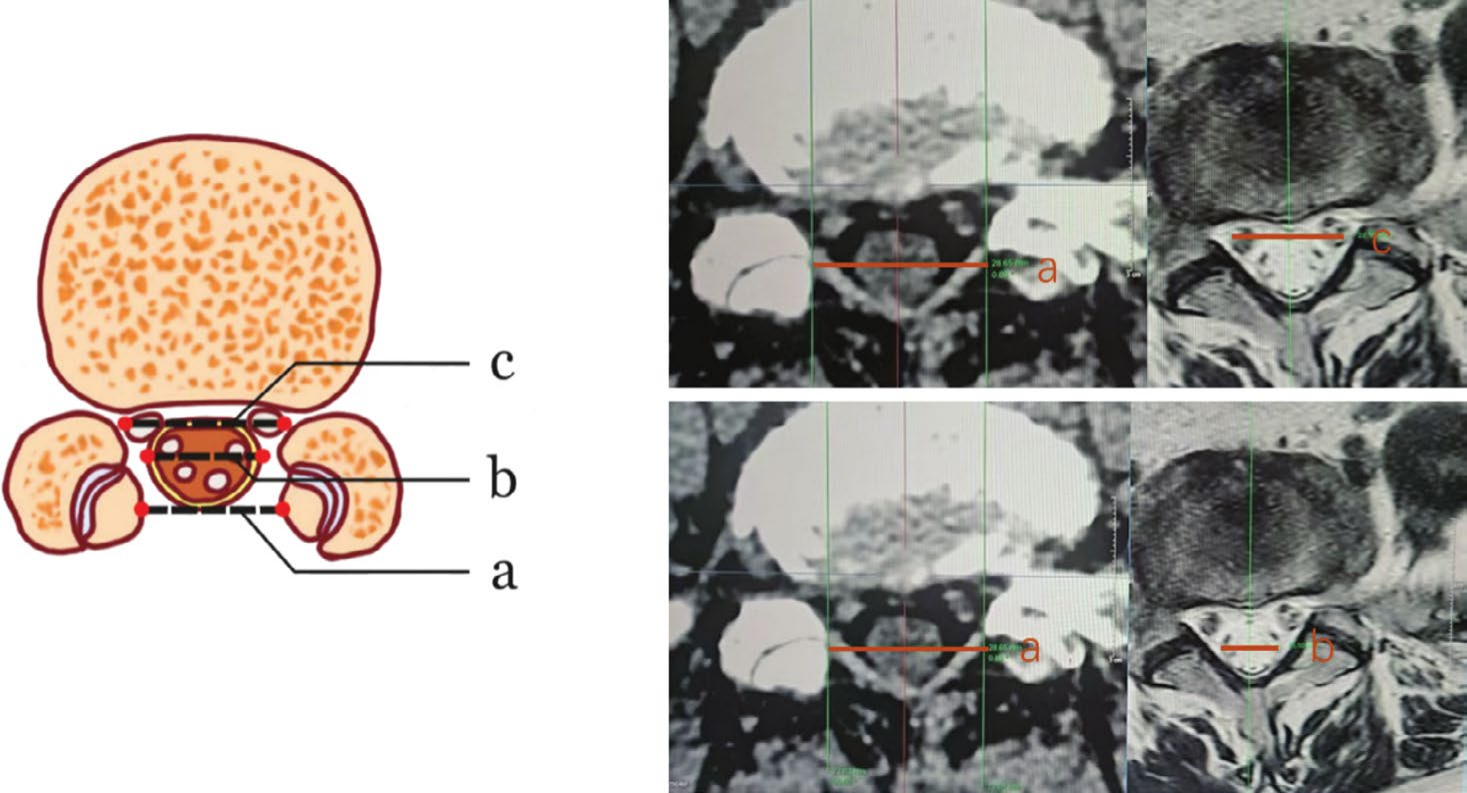
**a** Refers to the transverse diameter of the intersection point of the two sides (based on CT data), **b** refers to the transverse diameter of the dura mater at the same level (based on MRI), and **c** refers to the distance between the outer edges of the S1 nerve at the same level (based on MRI).

The half of the transverse diameter of bilateral puncture sites minus the transverse diameter of L5/S1 spinal dura sac (Figure 2).

$$\text{Dural}\;\mathrm{sac}\;\text{distance}=\frac{a-b}{2}$$

#### 2.4.2 S1 Nerve Distance

The half of the transverse diameter of bilateral puncture sites minus the transverse diameter of the lateral S1 nerves on both sides at the same level (Figure 2).

$$S1\;\text{nerve distance}=\frac{a-c}{2}$$

#### 2.4.3 L5 Nerve Distance

**FIGURE 3:**
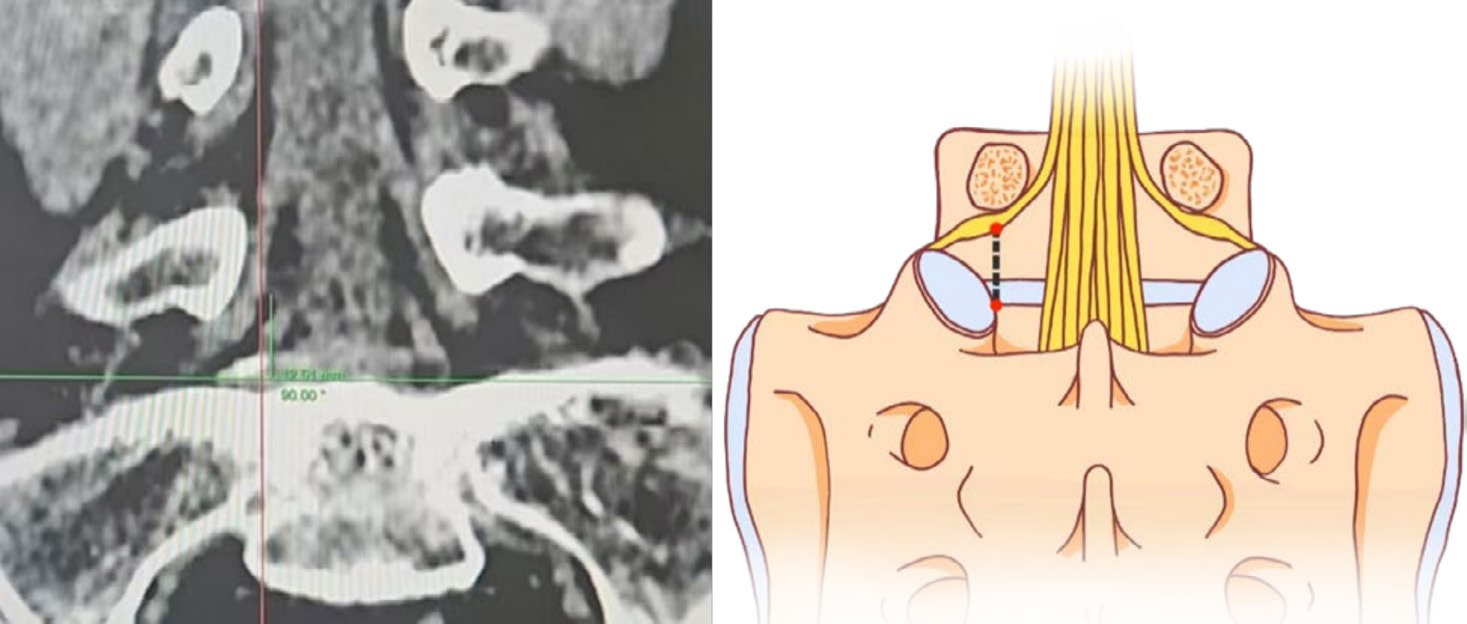
Distance between intersection point and L5 nerve.

The average of the distance between the inferior border of the L5 nerve and the intersection point measured in the vertical plane at the L5/S1 level on both sides (Figure 3).

#### 2.4.4 Vertebral Pedicle Distance

**FIGURE 4:**
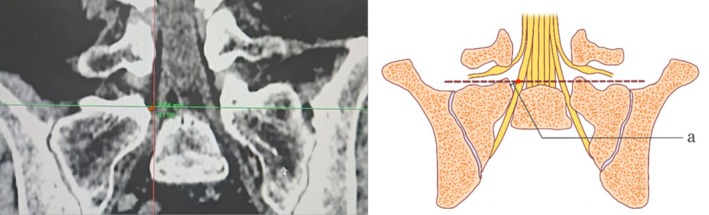
Distance between intersection point and inner edge of S1 pedicle.

**FIGURE 5:**
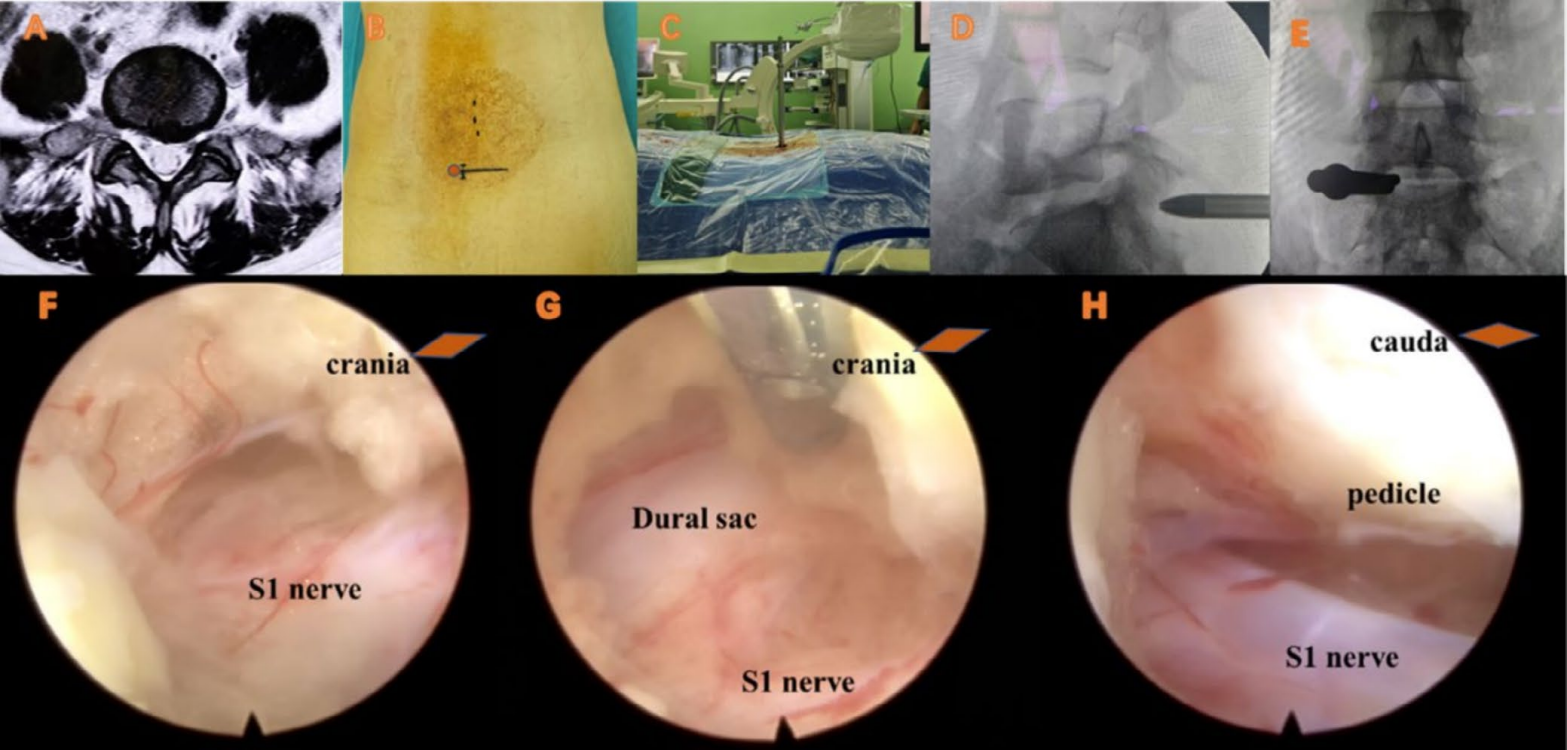
A young patient (age: 30–40 years old) presented with chronic radiating pain in the left lower extremity for 1 year, which had worsened for 1 month, and the operation time was 34 min. (A) Preoperative MRI suggested a left lumbar disc herniation at L5/S1. (B) The intersection of the superior endplate and the facet joint (orange dot) was used as the puncture target (facet joint degeneration was mild, and the incision was moved inward by 2 mm). (C) Insert the pencil tip vertically and establish the channel. (D, E) Fluoroscopy verification. (F) After removing a little ligamentum flavum, the vertically placed channel leads directly to the S1 nerve. (G) S1 nerve and dura sac were identified after cutting the ligamentum flavum. (H) S1 nerve and pedicle visible (flip lens).

The average of the shortest linear distance between the inner border of the vertebral pedicle and the intersection point measured in the horizontal plane at the L5/S1 level on both sides (Figures 4 and 5).

#### 2.4.5 Operation Time

From the time when the operative level was determined by fluoroscopy to the suture of the incision.

#### 2.4.6 Complication

Including dural tear, infection and so on.

Here, we represent the effectiveness by operative time and the safety by the occurrence of intraoperative complications.

### 2.5 Surgeon Selection

Two spinal surgeons with more than 5 years of experience in performing transforaminal lumbar interbody fusion had completed the training in PELD. They had not performed IELD surgery independently ever.

### 2.6 Statistical Analysis

Statistical analysis was performed using IBM SPSS Statistics for Windows, version 26.0 (IBMCorp., Armonk, N.Y., USA). Interrater and intra‐rater reliability were assessed with the intraclass correlation coefficient (ICC), respectively. All anatomic parameters are continuous data. We calculated the means and standard deviations (mean ± standard deviation). The operative time‐related learning curve was established using a cumulative sum (CUSUM) model. We used chi‐square tests for dichotomous variables and t tests for continuous variables. The logistic regression analysis was applied to exclude confounding factors.

## 3 Results

### 3.1 Radiographic Anatomy

The mean age of the included patients with CT and MRI data was 54.19 ± 19.23 years, including 46 males and 54 females (Table 1). The ICCs of all the radiographic measures were all above 0.8.

**TABLE 1:** Patient demographics.

Patient demographics	Outcomes
Age (mean ± SD)	54.19 ± 19.23
Sex (*n*)	Male: 46; Female: 54
Height (mean ± SD, cm)	171.34 ± 11.77
BMI (kg/m^2^)	25.22 ± 9.13
Dural sac distance (mean ± SD, mm)	4.59 ± 1.74
S1 nerve distance (mean ± SD, mm)	0.51 ± 0.91
L5 nerve distance (mean ± SD, mm)	10.14 ± 1.72
Vertebral pedicle distance (mean ± SD, mm)	3.77 ± 1.04
Learning period	15 cases, 17 cases
Dural tear	2/32 (in the learning period)
Surgical site infection	1/32 (in the learning period)

We found that the mean distance between the target and the dura sac was 4.59 ± 1.74 mm. After adjusting for age, sex, height, and BMI, logistic regression analysis showed that the main factor affecting the distance between the target and the dura sac was the patient's age (*B* = −0.51, 95% CI: −0.077, −0.033; *p* < 0.001).

The mean distance between the target and the inferior border of the L5 nerve was 10.14 ± 1.72 mm, rang from 7.52 to 13.54 mm. The mean distance between the target and the outer edge of the S1 nerve was 0.51 ± 0.91 mm, rang from −0.12 to 2 mm, and the target is almost located on the outer edge of the S1 nerve root.

The mean distance between the target and the inner edge of S1 pedicle was 3.77 ± 1.04 mm. After adjusting for age, sex, height, and BMI, logistic regression analysis showed that the main influencing factor was the patient's age (*B* = 0.033, 95% CI: 0.018, 0.058; *p* < 0.001).

## 4 CUSUM Analysis

**FIGURE 6:**
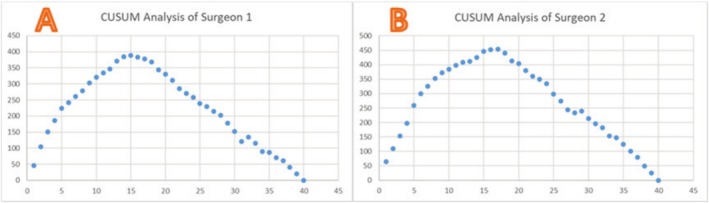
Learning curve of two surgeons.

The mean age of the patients included in the analysis for CUSUM analysis was 54.2 years. By CUSUM analysis, we found that the learning period of the two surgeons was 15 cases (Figure 6A) and 17 cases (Figure 6B), respectively. The average operation time in the mature period was 56.9 min, and the average operation time in the learning period was 100.78 min.

### 4.1 Complications

Both surgeons had one case of dural tear (2/32, 6.25%) in the learning period, but none in the mature period. One surgeon had one case of surgical site infection (1/32, 3.13%) in the learning period, but none in the mature period.

## 5 Discussion

IELD is a common surgical option for LDH, especially for L5/S1. However, how to select the puncture target and establish the working channel has lacked unified standards and specifications. In this study, we propose a standardized approach named the modified corner approach, which selects the intersection of the S1 superior endplate and facet joint as the target. Based on the radiographic analysis and clinical studies, we believe that the modified corner approach has the following advantages: 1. Standardization of operation. The intersection of the S1 superior endplate and facet joint was selected as the target or indicator, and the needle was inserted vertically. 2. Clear bony anatomical landmarks. The lamina boundary and facet joint could be seen outside the spinal canal, and the pedicle and vertebral endplate could be seen inside the spinal canal. 3. Safety. It directly reached the lamina when establishing the channel. 4. Convenience. The target is close to the outer edge of the S1 nerve root, and the shoulder and axillary decompression of the nerve can be easily achieved without excessive nerve traction. 5. Short learning period.

According to Ruetten [12] and Siepe [8], the IELD procedure can be divided into three steps. The first step is to establish the operation channel. In the second step, the ligamentum flavum is removed to expose the nerve and the herniated disc. In the third step, the herniated disc is exposed and removed, and the nerve is decompressed. Similar to other minimally invasive surgical modalities, IELD is limited by a narrow surgical field. Among them, the L5/S1 muscles are very abundant [13], and microscopic manipulation is often very difficult. Therefore, anatomical knowledge and spatial localization of the key structures are essential for the safe implementation of IELD.

IELD is a safe procedure with an overall complication rate of 11.2% [1]~18.1% [14]. The common complications include dural tear, infection, and inadequate discectomy [3]. The possible reasons are due to poor surgical technique and lack of understanding of the location of key anatomical structures [15]. With effective anatomical studies, useful information can be provided for preoperative planning and may lead to better outcomes and fewer complications.

Through the study, we found that the mean distance between the target and the L5 nerve was 10.14 mm, all of which were greater than 7.5 mm. The target was close to the outer edge of the S1 nerve, with a mean distance of about 0.51 mm. The mean distance between the target and the edge of the dura sac was 4.59 mm, and the mean distance between the target and the inner edge of the S1 pedicle was 3.77 mm. The radius of the tunnel commonly used in our clinic is 4 mm. Therefore, the modified corner approach has a good anatomical basis. On the basis of ensuring the visibility of the anatomical bone mark, all the surgical areas, including the S1 nerve root, are visible. In addition, it can accelerate the operation and reduce the risk of nerve injury by identifying the S1 nerve root and the dura sac in time. Meanwhile, these anatomical measures were not affected by gender and BMI, except for age. We believe it is mainly due to the influence of lumbar degeneration. In the older patients, the degeneration of facet joints was also more serious. The cohesion of facet joints was more serious, and the distance between the target and the dura sac was closer. Therefore, for patients without or with mild facet joint degeneration, the puncture target can be appropriately moved inward by 2 mm.

The radiographic measures were obtained in the supine position, while during the operation, the patient's position was prone. This may affect the validity and practical significance of our study conclusions. Kinetic MRI (kMRI) can image patients in a weight‐bearing position (either standing up or sitting) and in flexed and extended positions, thus revealing abnormalities that are missed by traditional MRI studies [16]. In the previous literature, kMRI revealed changes in lumbar spine alignment in different positions [17]. The results of Tan et al. [18] showed that in kMRI for patients with Pfirrmann grade I or II, the mean movement distance and angle of the L5/S1 segment were 1.01 mm and 5.97°, which were significantly lower than other lumbar segments. This means that the L5/S1 segment has the least range of motion in different positions. In our study, the most likely affected index in different positions was the distance between the target and the L5 nerve root. But the results suggested that the mean distance between the target and the inferior border of the L5 nerve was 10.14 ± 1.72 mm, ranging from 7.52 to 13.54 mm, which was much larger than the radius of the endoscope. Therefore, the changes in spinal alignment in different positions had little effect on the results of this study.

Another noteworthy point of the IELD procedure is the long learning curve [7]. The meta‐analysis of Ahn et al. [7] suggested that the average learning period of IELD was 22 cases. In Josiwig's clinical study [19], the mean value for the cutoff point was 22.17 ± 12.40 cases (range: 10–43 cases). In our study, we included a total of 80 IELD cases with two surgeons. We found that procedure times leveled off after 15 and 17 cases for both surgeons, respectively, and neither had a single postoperative complication in the mature period. IELD based on the modified corner approach can accelerate the learning speed of young surgeons and reduce the difficulty and risks of surgery. Meanwhile, the complications of IELD based on the modified corner approach were also rare. The results indicate that IELD based on the modified corner approach is safe and effective.

### 5.1 Limitations

Firstly, there may be some differences between radiographic anatomy and cadaveric dissection. Secondly, anatomical measures were obtained from the CT or MRI data without nerve compression, while surgical patients will have nerve compression. Therefore, the application of the results to real‐world patients' needs to be cautious, and attention should be paid to anatomical changes caused by positional changes and degenerative changes in reality.

### 5.2 Clinical Application and Prospect

The modified corner approach provides a standardized and reproducible surgical pathway. Preoperative imaging clearly delineates the spatial relationships between the herniated disc, nerve root, and dura mater in patients with LDH. Utilizing this approach, when the working channel is positioned perpendicular to the floor, the relative position of these critical structures—the nerve root, dura mater, and herniated disc fragment—can be readily identified within the working channel. This enhanced visualization facilitates faster surgery, reduces procedural complexity, and lowers the risk of intraoperative complications.

## 6 Conclusion

The modified corner approach is a simple, standard, and repeatable surgical approach with the intersection of the S1 superior endplate and facet joint as the puncture target. Based on the radiographic data, we obtained the relative positions of the inner edge of the pedicle, the outer edge of the dura sac, the S1 nerve, and the L5 nerve between the target in the modified corner approach. The anatomy can be affected by the age of the patient. For patients without or with mild facet joint degeneration, the puncture target can be appropriately moved inward by 2 mm. By analyzing the IELD operative learning curve of the modified corner approach, we proved the anatomical basis and clinical significance of the modified corner approach.

## Author Contributions

Sizheng Zhan, Xiangsheng Tang, and Ping Yi were responsible for research design. Haoning Ma and Sizheng Zhan collected and analyzed the patient clinical and hematological data. Sizheng Zhan and Haoning Ma were major contributors to writing the manuscript and should be listed as co‐first authors. Haoning Ma and Yuming Wang are the two surgeons selected. All authors read and approved the final manuscript.

## Disclosure

Sizheng Zhan, Haoning Ma, Yuming Wang, Ping Yi, and Xiangsheng Tang declare that all authors listed meet the authorship criteria according to the latest guidelines of the International Committee of Medical Journal Editors and that all authors are in agreement with the manuscript.

## Ethics Statement

The study was approved by the Ethics Committee of China‐Japan Friendship Hospital (Approval No. 2024‐KY‐263). Informed consent was obtained from all participants prior to their inclusion in the study. This study was conducted in accordance with the principles of the Declaration of Helsinki.

## Consent

The authors have nothing to report.

## Conflicts of Interest

The authors declare no conflicts of interest.

## Data Availability

Data sharing not applicable to this article as no datasets were generated or analysed during the current study.
